# Guidance for management competency identification and development in the health context: a systematic scoping review

**DOI:** 10.1186/s12913-023-09404-9

**Published:** 2023-05-01

**Authors:** Edris Kakemam, Zhanming Liang

**Affiliations:** 1 Clinical Research Development Unit of Tabriz Valiasr Hospital, Tabriz University of Medical Sciences, Tabriz, Iran; 2 College of Public Health, Medical and Veterinary Science, James Cook University, Townsville, Australia

**Keywords:** Management competence, Competency development, Competency-based framework, Health manager, Competency identification

## Abstract

**Background:**

Using management competency-based frameworks to guide developing and delivering training and formal education to managers has been increasingly recognized as a key strategy in building management capacity. Hence, interest in identifying and confirming the competency requirements in various contexts have been witnessed. Therefore, learnings from how competency studies were designed and conducted, how competencies were identified, and strategies in ensuring success in competency identification are of great value to researchers planning and conducting competency studies in their own country.

**Methods:**

A scoping review was conducted guided by the Arksey and O’Malley framework and reported according to the PRISMA Extension for Scoping Reviews (PRISMA-ScR). All papers that published empirical studies aiming at identifying and assessing manager’s competencies at the peer-reviewed journals were identified from Web of sciences, PubMed, Scopus and Emerald Management between 2000 and 2021. In order to maximize learning, studies focusing on health and non-health sectors are all included.

**Results:**

In total, 186 studies were included in the review including slightly more than half of the studies conducted in health sector (54.5%). 60% of the studies focused on mid to senior level managers. Surveys and Interviews were the two most commonly used methods either solely or as part of the mix-method in the studies. Half of the studies used mixed methods approach (51.1%). Large proportion of the papers failed to include all information that is necessary to contribute to learning and improvement in future study design. Based on the results of the scoping review a four steps framework was developed that can guide designing and implementing management competency studies in specific country vs. sector context and to ensure benefits of the studies are maximised.

**Conclusion:**

The review confirmed the increasing trend in investing in management competency studies and that the management competency identification and development process varied substantially, in the choice of methods and processes. The identification of missing information in majority of the published studies calls for the development of more rigorous guidelines for the peer-review process of journal publications. The proposed framework of improving the quality and impact of the future management competency study provides clear guidance to management competency identification and development that promotes the functional alignment of methods and strategies with intended uses and contexts.

**Supplementary Information:**

The online version contains supplementary material available at 10.1186/s12913-023-09404-9.

## Background

In recent decades developing a competent health service management workforce has been well recognized as one of the means to improving health service effectiveness and efficiencies [1–3] which is critical to the sustainability of health systems in order to meeting the increasingly complex health needs and demands. Therefore, global movement in developing better understanding of the competencies required by health service managers in different healthcare context (countries vs. settings vs. management positions) has been witnessed [4–8]. The importance of adopting a competency-based approach in designing and developing training and educational programs in health management has been recognized as the means to building health system management capacity and improving effectiveness and quality of health service delivery [2, 9–12], making the understanding of management competency requirements for health service management fundamental [2, 13].

Management competencies are context-sensitive and influenced by the complexity of management levels, nature of management positions, and the team, organization and system in which these competencies to be demonstrated. No one healthcare system is the same, differing healthcare environment and health policy of different countries may affect the competency requirements of their managers differently. Hence, management competencies cannot be adopted without considering the differing healthcare context [14–17]. In the past two decades, increasing number of management competency studies have been undertaken in the developed countries such as Australia, USA, UK, Canada, Switzerland and Finland [1, 5, 8, 11, 18, 19]. There has also been increasing reinforcement of the importance of health management competency in developing countries such as Iran, Thailand, Vietnam, South Africa, Bhutan, and Indonesia [4, 7, 9, 20–22]. Although efforts in developing country-based understanding of the management competency requirements have been continuous and increasing, in comparison with the efforts in non-health sector, management competency development in health sectors is still in its infancy, hence learning the experience of management competency identification from both health and non-health sectors are beneficial.

In addition to overcoming common challenges facing any research projects, the identification of management competency requirements have its own hurdles to overcome and can be a complex process. Firstly, the concept and role of management competency have been interpreted differently between researchers and managers. Bridging such understanding is critical to ensuring the relevance and validity of the competency identified. Secondly, health service management has not be universally accepted as a certified profession and represents a large number of different positions varied by management level, functionality and settings [2] which makes creating a ‘blanket competency framework’ less straightorward. Thirdly, the recruitment and involvement of health service managers in management competency studies are challenging because of the busy nature and associating high pressure of the management roles, often resulting in low response [2]. Fourthly, the validity of the process of identifying management competency is important to ensuring that the competency framework developed can guide formulation of management training curriculum. Such process is also important to confirm the context in which the developed management competency framework can be used to guide application.

While no guidance is currently available on what specific methods to use, and when and how to use to identify management competencies, there is consensus that in order to increase the validity and utility of competency frameworks developed, a combination of approaches may be necessary, akin to a process of triangulation [23]. However, consideration of the feasibility of the study, complexity of practice, and ease of access to appropriate stakeholders may prompt research designers to prioritise aspects of the competency development and validation process differently [23, 24]. These challenges may result in variable or uncertain study outcomes that may limit the validity and applicability of the research output as a result [23, 24]. Therefore, experience and lessons learnt from previous management competency studies would be beneficial in guiding future management competency studies in determining research resign and data collection methods and processes. Such learning will contribute to the successful implementation and completion of the studies by incorporating tested strategies in the study design. In addition, such learnings can also provide guidance to increase the applicability and validity of the management competency framework or management competency tool developed. It is also reasonable to assume that using tested methods can decrease the time and cost associating with new competency framework development and maximize the research efforts.

With such purposes in mind, a scoping review on empirical management competency studies was conducted in 2021 in order to answer the following questions:

1. What are the commonly used methods in identifying management competencies? Are there specific considerations on how different methods could achieve competency identification purposes?
2. What are the key learnings and specific strategies adopted that can contribute to the success in management competency studies?

The findings of the review provide guidance for determining the design and implementation of the management competency studies and the application of the identified management competencies.

## Methods

### Study Design

The scoping review was completed following the five-step process described by Arksey and O’Malley [25] and Levac et al. [26] and reported according to the PRISMA Extension for Scoping Reviews (PRISMA-ScR) [27] and conducted by the authors EK and ZL, and a colleague AH. The review includes studies in both health and non-health context for broader learning.

#### *Paper inclusion criteria*

- Presented results of empirical studies and systematic / scoping / rapid review;
- Are published in English language since 2000 considering the commencement some major management competency reviews and studies since 2000, and.
- Are published in a peer-reviewed journal that focus on management competency identification and management competency assessment.

#### *Paper exclusion criteria*

- Opinion pieces and commentaries without linking to empirical evidence,
- Are not published in English,
- Are not published in peer reviewed journals, and.
- Were published prior to year 2000.
- Papers presented results of systematic / scoping / rapid reviews were excluded from data extraction.

### Data source and search strategy

The search strategies were developed by the authors who have both content knowledge and experience in conducting scoping review. Searches in databases were conducted in December 2019 using the following key words or combination of the key words: “competenc*”, “leader*” “head nurses”, “charge nurses”, “directors”, “executives” and “manage*”, including literature from both the health and non-health sectors. Electronic databases such as PubMed, Scopus, Web of Science and Emerald where majority of the management studies are published were initially accessed. An additional snowball search technique such as scanning reference lists to identifying relevant papers was also applied to compliment the search. Details of the search strategies using terms and combination of terms were explained in “Supplementary 1”.

### Screening

Search results were imported into the EndNote software (V.X8) with duplications removed. Titles and abstracts of the articles generated by the searches were screened independently by EK and AH leading to the confirmation of potentially relevant articles. Once agreement was reached between EK and AH, full-text review of the articles were performed by EK in accordance with the inclusion criteria. The final decision on papers to be included in data extraction was made on the basis of agreement between EK and AH. Views from ZL were sought to resolve disagreement.

### Selection of studies and data extraction

Data were extracted independently by EK and AH from the selected studies using data extraction form developed and pilot tested for each study design. The following information were extracted and entered into the excel spreadsheet prior to the analysis: (A) surname of the first author, (B) year of publication, (C) country of origin, (D) period of data collection, (E) study aims; (F) methods used to develop and identify management competency (G) settings in which studies were conducted, and (H) main findings.

### Critical appraisal

In line with the scoping review framework, we did not conduct a critical appraisal on the papers [25].

### Data charting and analysis

A data extraction form was developed to organize information, confirm relevance, and to extract study characteristics from included articles. Information collected included study characteristics, objectives of studies, and citations. Relevance was confirmed by sampling population and objectives. Characteristics collected via this form included: author (year), country; sampling population; objective/aim of studies; setting (health sector or non-health sector); methods used; management level. All data were compiled into a single spreadsheet in Microsoft Excel 2013 (Microsoft, Redmond, WA) for coding and analysis.

## Results

### Study selection

**Fig. 1:**
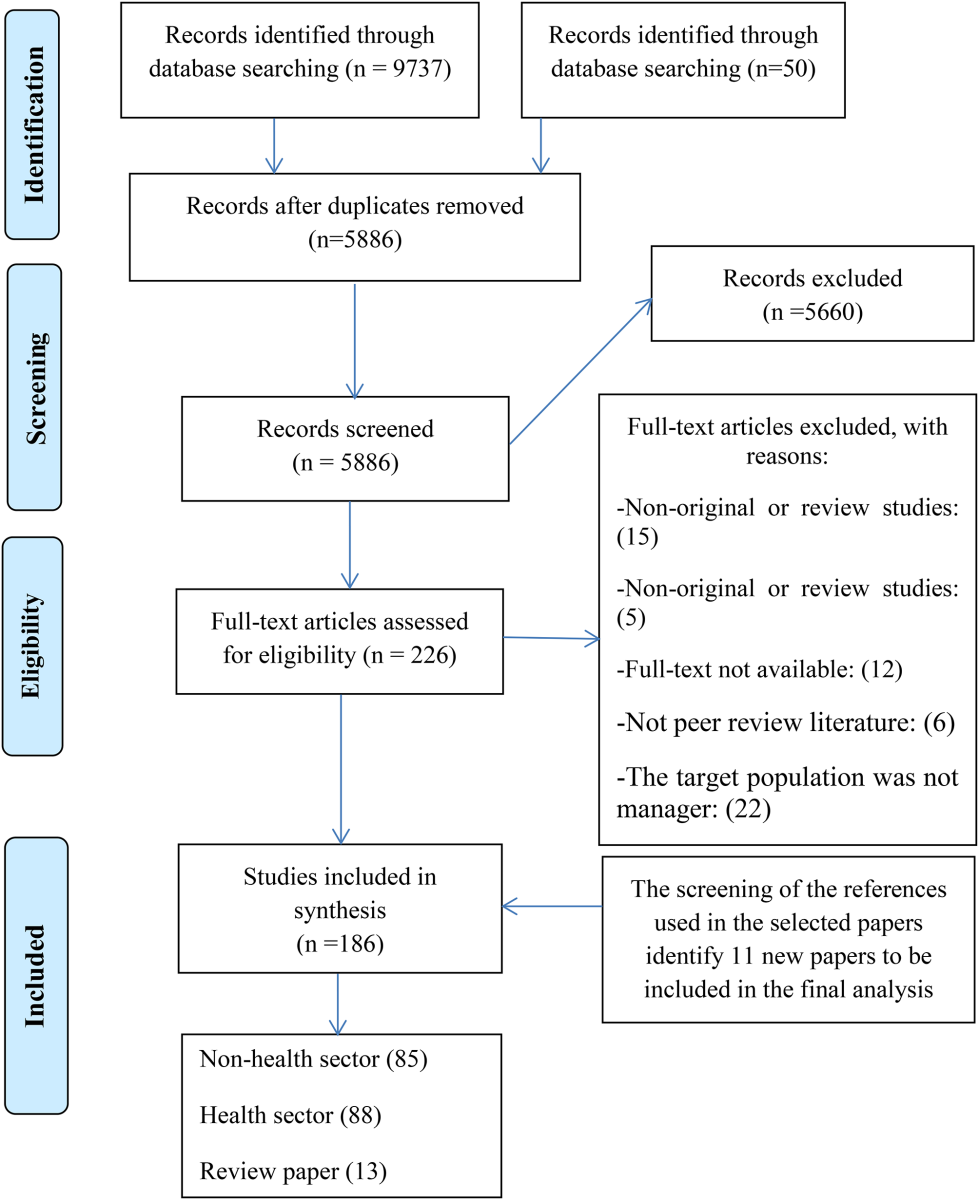
Search and review strategy (PRISMA flow diagram)

The review process and search results are presented in Fig. 1. In the initial search, 5936 articles were found in the four selected databases by keyword search as explained earlier. Abstract screening resulted in 226 studies being included for full-text review. As a result, 173 studies (excluding 13 review papers) published between 2001 and 2021 were included in data extraction and analysis.

### Study location

**Fig. 2:**
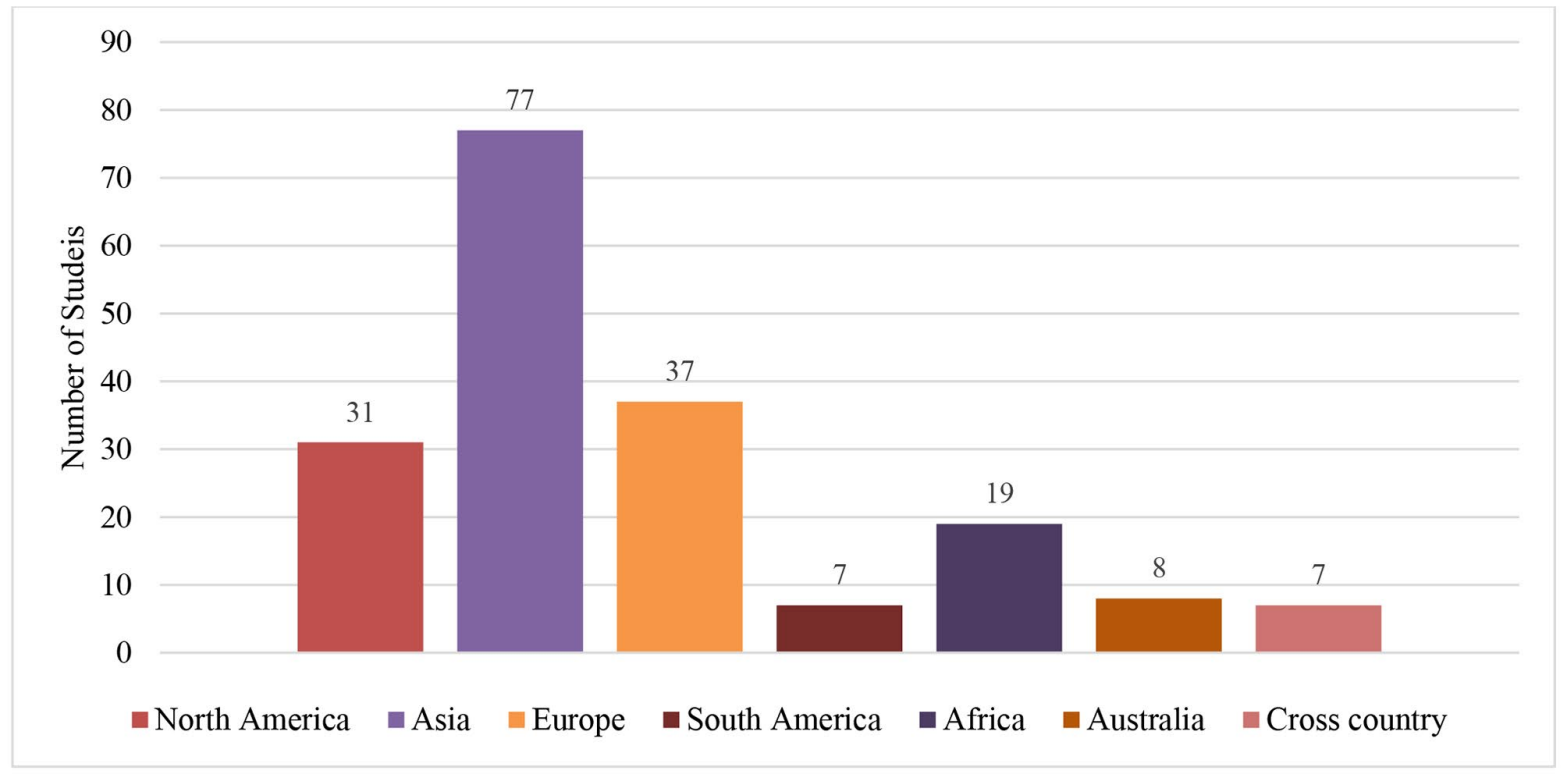
Study location and number of studies

Location of the studies conducted is presented in Fig. 2: 41.4% of the studies were conducted in Asia (n = 77), followed by Europe (19.9%, n = 37) and North America (16.7%, n = 31).

**Fig. 3:**
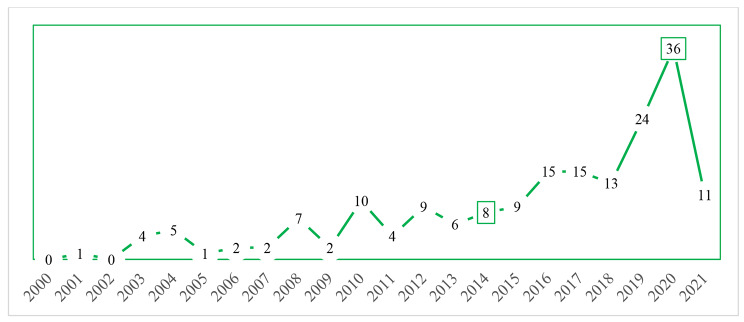
Published year and number of studies

Most of the 186 studies were published in 2019 and 2020. Figure 3 shows an increasing trend in the publication of management competency study, in particular the significant increase since 2019.

### Sectors and target population

Amongst the 173 empirical studies, 88 vs. 85 papers presented the studies that were conducted in health and non-health sectors respectively. Target population varied between management levels, management positions and sectors. For studies in the health sectors, close to half of them focused solely on senior or middle management levels or both levels (n = 44); 27 of them on junior or both middle and junior levels, and 17 of them targeted various management levels. On the other hand, for studies conducted in the non-health sectors, 66 out of 85 studies (77%) solely focused on senior and middle management level. The rest were on junior levels and all management levels.

### Methods used to identify management competency

Half of the studies (n = 92) adopted mix-methods for data collection. The other 84 studies adopted single-method. Amongst the single-method studies, 45 vs. 36 used quantitative and qualitative approach respectively. Table 1 details the frequency of the eight methods used by the 173 papers (excluding the 13 review papers). The commonest methods used were survey and interviews.

**Table 1:** Methods used by management competency studies

Methods/strategies	Number of studies	Percentage of all 173 studies identified
Survey	110	63%
Interviews	59	43%
Delphi technique	27	Less than 16%
Focus Group Discussions	19	
Document analysis	13	
Expert panel	17	
Observation	1	

### Variation in application of methods/ strategies

Considering the popularity of ‘surveys’ and ‘interviews’ which dominated majority of the management competency studies both in health and non-health sectors, the paper focuses on the actual learnings from these two methods. Learnings include the considerations and preparation put into designing and conducting the studies, and whether different study outcomes in terms of competency identification and development were expected as a result.

To achieve such purposes, the second round of data extraction was performed from studies adopted surveys and interviews as part of the research methods. The data extracted included.

- Justification of methods chosen;
- Sampling frame, sampling methods and recruitment strategies;
- Sampling power, and.
- Discussions of the generalisibity of results and/or results implications.

### Learnings from surveys

Survey was the commonest method used by 110 out of 173 identified studies as either sole method for 44 studies or part of the mix-methods for 66 studies for four main purposes:

1. To confirm competencies identified in other studies and/or test the applicability in their own context;
2. To assess competency level of managers within specific context;
3. To identify core competencies for specific management positions, and.
4. To obtain feedback during the competency identification and competency framework development process.

Overall, 46% of surveys were conducted paper-based (n = 51) and 39% was conducted electronically either using an online platform or via email (n = 43). Ten surveys were conducted both online and face to face [9, 28–36]. Six articles did not mention how survey was conducted. Few students opted to conduct the survey on paper because of the concern of low response for online method.

Slightly more than half of the identified studies (n = 59) did not mention methods used to recruit survey participants. Thirty-six studies invited the whole target population for participation and the rest of study selected study participants using two main techniques namely probability sampling (n = 24) and non-probability sampling (n = 23). Random sampling (n = 16) and convenient sampling (n = 11) are the two commonly used sampling methods. Purposeful and snowball sampling was mentioned by 13 studies. Two studies used Census approach and plausibility sampling. One study used two methods purposeful and stratified sampling simultaneously.

All surveys included closed and structured questions to construct questionnaires. Most of the questionnaires used Likert scale with 5-point scale being the commonest (n = 65 or 59.1%). Studies did not normally provide clear justifications of how questionnaire was constructed, why certain Likert scale was used and whether such scale has been validated in studies with similar nature. Survey instrument was pilot tested in 37 of the studies prior to the commencement of the survey.

Sample size greatly varied between studies. Table 2 details the number of studies falls into different sample size ranges.

**Table 2:** Number of participants recruited in survey

Number of Participants	Number of Studies	% of identified studies
Less than 100	34	31%
100–299	53	48%
300–500	13	12%
More than 500	10	9%

However, studies generally did not discuss how sample size affected the applicability and generalizability of the results, even the five studies with a response rate lower than 20%. Amongst the 92 studies using survey as one of the mix-methods, 1/3 of them did not report actual response rate. For the rest of the studies, the mean response rate is 69% ranging from 8% [37] to 100% [38–44] including.

- 25 studies achieved a response rate of 90% or higher [4, 7, 9, 28, 34, 35, 38–56].
- 33 studies achieved a response rate between 50 and 89.9% [2, 30, 57–87], and.
- 6 studies with a response rate less than 20% [37, 88–92].

Only 25% of the papers (n = 27) mentioned or discussed recruitment strategies as the following:

- Be flexible allowing paper-based survey to supplement the online survey when online survey response rate is lower than planned;
- Having senior leader to reinforce the importance of the study and encourage participation from their staff;
- Having the survey open for longer period ( 4 weeks or longer) with multiple reminders via emails or phone calls;
- Identifying a contact person from each participating organization to assist with the recruitment of survey participants and survey distribution. The contact person usually are senior manager or director of human resources;
- Minimizing the complexity and length of the questionnaire used for the survey;
- Mobilizing own professional and social network to recruit additional eligible participants;
- Recruiting and training clinical staff such as nurses to assist with the implementation of paper-based survey including distribution of questionnaires and collection of the completed questionnaires, and.
- Reinforcing the anonymous and confidential nature of the survey.

### Learning from interviews

In total, 59 out of the 173 studies (34%) used interview as a sole method or part of the mixed method for data collection for the following key purposes:

- To develop rich understanding of management behavior and performance [4, 9, 20, 29, 36, 37, 39, 54, 58, 61, 63, 66, 74, 79, 80, 86, 93–109];
- To identify managerial competencies and behaviors that can predict job performance [10–12, 20, 39, 54, 58, 61, 66, 74, 80, 94, 98, 104, 106, 108, 110–130];
- To develop rich understanding of the findings generated from the previous step(s) of the same study [29, 36, 63, 86, 96, 97, 99–102, 107, 109], and.
- To gain insight into actual management practices from both managers and stakeholders’ perspectives [4, 9, 37, 79, 93, 95, 103, 105, 131, 132].

Interviews were most commonly designed as in-depth and semi-structured [9–11, 36, 37, 39, 58, 61, 93–96, 100, 101, 103, 106, 109, 111, 114, 120–124, 128–130, 132]. Critical incident interviews [80, 102, 112, 113, 128] and behavioral event interviews [12, 86, 105, 116, 127, 131] were also performed. Interviews were conducted online [115], by telephone [63, 98, 115], and face-to-face [10, 11, 39, 58, 79, 86, 109, 114, 120, 125], both online and face-to-face [9, 129], or both telephone and face-to-face [36, 122]. Nine studies did not explicitly outline the form of interviews [20, 66, 74, 97, 103, 104, 107, 108, 126]. Online interviews were conducted in the studies published in 2012, 2019 and 2021.

Several studies (n = 16 studies) mentioned that protocol including pre-determined open questions was developed to guide the interview process. Less than 40% of the studies (23 out of 69) provided adequate details on how interview questions were developed [11, 12, 29, 61, 86, 93–96, 98, 99, 103, 109, 111, 115, 117, 121, 123, 124, 129, 131, 132]. The following common methods were used to develop interview questions:

- Adopted an interview guide from other studies, then validated with representatives of target group and made adjustment before implementation, and.
- Conducting document analysis on policy and government documents and reports;
- Conducting literature review to identify competencies from other studies;
- Developed by investigators, then input sought from professional institutions that provide training to the target population;
- Developing questions with inputs from representatives from target population;
- Informed by findings of the quantitative data collected in the first phase of the study, and.
- Using the critical incident technique to gather information about effective and ineffective individual behaviors.

The number of interview participants varied between studies. Forty-two studies interviewed between 10 and 35 participants. Five studies involved more than 35 interviewees (n = 36, 42, 45, 110 and 120 participants) [11, 111, 115, 124, 128]. Eight studies conducted interviews with less than 10 participants [9, 29, 54, 61, 102, 103, 107, 129] including three studies only interviewed three or four participants [9, 54, 129]. Most of the studies did not provide justification of why specific number of interviewees were recruited for the interviews. However, theoretical saturation was one justification mentioned in eight studies.

## Discussion

The increasing interest and investment in management competency studies in the past five years confirms the value of the development management competency framework which can serve as valuable resources for guiding the design of training and education curriculum, outlining requirements of a competent workforce and professional development needs, evaluation or assessment of professional expertise [2, 133–136]. It also confirms that ongoing efforts in refreshing and modifying management competency-based frameworks are required in order to respond to the changing environment or professional needs, minimize lapses in knowledge and skill sets, and ultimately transform education for professions [137]. The increasing interests in management competency studies align with the increasing reinforcement of the role of management competency in service quality and efficiency improvement. The sudden surge of publications in 2020 at the peak of COVID-19 pandemic reinforced the importance of ensuring management competency and capacity development being responsive to the pressing needs of improving health service quality and efficiency.

The aging of the health workforce and the changing healthcare needs and complexity making the delay in effective health workforce development in terms of current workforce upskilling and producing ‘new blood’ impossible [138]. It has been recognised that, in addition to playing a key role in improving service quality and efficiency, managers is critical to leading and managing healthcare transition, and the adoption of innovation is no longer an option, but a most, in order to creating a sustainable healthcare system [139]. The drastic increase in the number of management competency studies conducted in developing countries, in particularly in Asia in the past 10 years proven the necessity of considering learning from past management competency studies, in particularly in the healthcare sectors, when designing its own management competency studies to improve efficiency.

Disregard whether study adopts a single method or mix-methods or using surveys and interviews in data collection, ‘validating’ findings (identified management competencies) from previous studies should be considered to avoid duplication or ‘reinventing the wheel to create something new’. This is backed up by ‘the existence of core management competencies across management levels and sectors’ as suggested in the management literature [13]. The review of previous studies confirmed that ‘validation’ can be considered by both qualitative and quantitative method such interviews and surveys. Questions in survey can be drawn from the findings of management competency studies whilst interview questions were often developed based on prior knowledge which can be gained from literature review, document analysis and validated interview questions from previous studies. Key stakeholder engagement and consultation was also one key strategy in seeking input from target group and professional bodies.

The review confirms the importance of adopting a flexible approach for conducting management competency studies considering the time constraint and high workload demands of health managers. These include the mixture of conducing the questionnaire survey both online and on paper creating flexibility for busy health managers in completing the questionnaire whenever they have a spare moment. COVID-pandemic has changed how health services are provided and how works are completed bounded by the constraint of travel restriction, cross-infection prevention and maintaining social distancing [140]. E-Health, online consultation, holding meetings via online platforms such as zoom have become regular part of our lives [141]. Therefore, it can be predicted that online interviews, online focus groups etc. would be considered as a common process for research data collection. This posted a question of whether qualitative researchers have been equipped with the relevant skills to conduct qualitative studies via online platform where body language was hard to observe and participant engagement is harder to be effective.

Learning from previous studies, survey and interview can contribute to achieving the purposes of the studies differently. Surveys can be used to validate and identify management competency requirements, whilst interviews placed focus on linking management competencies to actual demonstration and performance. Interviews also helped developing understanding of the context in which that management competencies are applied. Management competencies are context sensitive [134, 135], competencies identified via quantitative methods are helpful in developing a guiding framework for understanding competency requirements and development needs. But it is the qualitative method that helps to understand the context in which the competency framework is most applicable to and in what ways that the framework should be used to guide competency development. The popularity of mix-methods used by the identified studies confirms the multiple purposes.

One of the key challenges for conducting management competency studies using quantitative method is successful participant recruitment in achieving a response rate allowing the generalization of result. The identified studies confirmed two important strategies: getting support/buy in from participating organisations and adopting a flexible approach as discussed above. Identifying a liaison person who has credibility and good access to targeted management positions, senior management’s openly providing support to the study, and training nursing staff in providing actual assistance in administering the questionnaire survey are three key strategies. The validity and reliability of the chosen method, the practicability and acceptability to relevant stakeholders including potential research participants are also the considered factors [142]. All of these require advanced planning and relationship building.

**Fig. 4:**
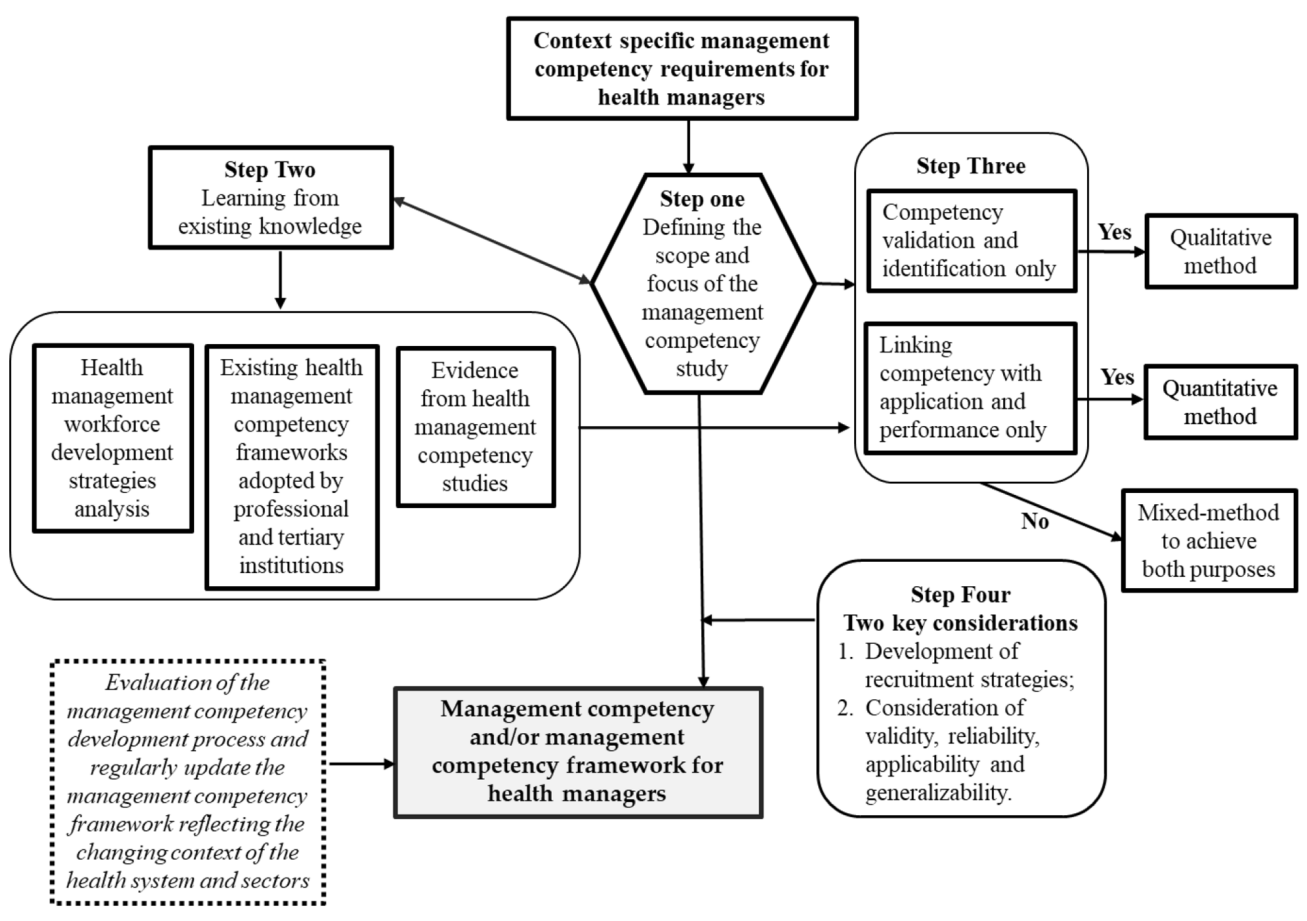
Competency framework development of management in healthcare

Based on the results of the scoping review and the health management competency identification process validated by Liang et al. (2018) [5], the below framework (Fig. 4) is developed that can guide designing and implementing management competency studies in specific country vs. sector context and to ensure benefits of the studies are maximised.

The first step in a competency modeling effort is to define the scope and objectives of the study, which includes considering the opportunities to be pursued through the development and application of a competency model. This step is also informed by ‘prior learning (step two) and informs the determination of research design and method for data collection (step three). It is important to consider how will the identified competencies and competency framework be used to guide strategic workforce planning, employee selection, performance management, training and development, succession planning, rewards and recognition, or career planning. The success of management competency studies also rely on the participation and active contribution make by the managers. Therefore, it is important that the ‘two key considerations’ (Step four) are incorporating into the research project design and implementation. Competency requirement for health managers continues to revolve as a response to the rapid transformation of the healthcare system. Efforts in regularly review and update the competency requirements is necessary to ensure the relevance and applicability of the management competency frameworks developed.

The review also identified information critical to study success and accessing the validity and reliability of the study are consistently missing inform the published management competency studies. The information, such as process of constructing survey questions and selecting target population, and recruitment strategies, should be included in research reports and published journal articles as they can allow learning from others to improve future research efficiency and success.

The review found that discussion on how sample size affected the applicability and generalizability of the results and the context in which management competencies are applicable were also often missing. This calls for the review and development of more rigorous guidelines for article peer-reviewed process by journals acting as gate keeper for quality publications and allowing not only the sharing of research results and discussing implications of the studies, but also encouraging lessons to be shared and leant contributing to better research design and research efficiency.

### Strengths and Limitations

A strength of this scoping review is that the search strategy was comprehensive with assistance of a skilled research librarian. In addition, a rigorous process was used to determine which articles should be included in the final review. The review was conducted according to PRISMA-ScR guidance. However, there are some limitations of our research that may affect the results and should be addressed. First, quality of the articles was not assessed. Second, this review was restricted to peer-reviewed English papers and did not including books, grey literature, or any documents published in a foreign language. As a result, some evidence might have been excluded. However, this does not inherently bias a review [143].

## Conclusions

The development and the use of competency is a complex endeavor. This review identified and explored publications sharing results of management competency identification and management competency framework development in both health and non-health sectors. The review confirmed an increasing trend in investing in management competency study and that the management competency identification and development process varied substantially, in the choice of methods and reporting of the process. Despite the expectations of comprehensive presentation and discussion of study design, implementation strategies and applicability of the research results, some important information are missing from large number of the identified studies limited the learning from experience that is important to improve future research design and research quality. The proposed framework of improving the quality and impact of future management competency studies provides a clear guidance to the process of management competency identification and development that promotes the functional alignment of methods and strategies with intended use and context. In addition, such guidance will assist researchers in determining approaches better-positioned studies to overcome challenges associated with competency framework development.

## Electronic supplementary material

Below is the link to the electronic supplementary material.

Supplementary Material 1

## Data Availability

The datasets used and/or analysed during the current study available from the corresponding author on reasonable request.

## Abbreviations

PRISMA-ScR: PRISMA Extension for Scoping Reviews
