# Supplementary material for: Guidance for management competency identification and development in the health context: a systematic scoping review
Source: BMC Health Serv Res. 2023 May 1;23:421. doi: 10.1186/s12913-023-09404-9 (PMC10150671; doi:10.1186/s12913-023-09404-9)
Supplement: Supplementary file 1 — Supplementary Material 1 [file 12913_2023_9404_MOESM1_ESM.docx]

Supplementary 1. PubMed, WOS, Scopus and Emerald search strategies (1 May 2021)

| **Database** | **Search term** | **Results** |
| --- | --- | --- |
| **PubMed** | ("manage*"[Title] OR "leader*"[Title] OR "head nurses"[Title] OR "charge nurses"[Title] OR "directors"[Title] OR "executives"[Title]) AND "competenc*"[Title/Abstract]Limit to ≥2000 | 3002 |
| **Scopus** | ( TITLE ( manage* ) OR TITLE ( leader* ) OR TITLE ( directors ) OR TITLE ( executives ) OR TITLE ( "charge nurse" ) OR TITLE ( "head nurses" ) AND TITLE ( competenc* ) ) )  AND  PUBYEAR  >  1999 | 2290 |
| **WOS/ISI** | TITLE: (manage*) OR TITLE: (leader*) OR TITLE: ("head nurses") OR TITLE:  ("charge nurses") OR TITLE: (directors) OR TITLE: (executives) AND TITLE:  (competenc*) Refined by: PUBLICATION YEARS: ≥2000 | 1655 |
| **Emerald** | title:"competenc*" AND (title:"manager") OR (title:"leaders") OR (title:"directors") OR (title:"executives") Limit to ≥2000 | 2790 |
